# Barriers and Facilitators for Exclusive Breastfeeding in Women’s Biopsychosocial Spheres According to Primary Care Midwives in Tenerife (Canary Islands, Spain)

**DOI:** 10.3390/ijerph18073819

**Published:** 2021-04-06

**Authors:** Seila Llorente-Pulido, Estefanía Custodio, Maria Rosario López-Giménez, Belén Sanz-Barbero, Laura Otero-García

**Affiliations:** 1 Servicio Canario de Salud. Gerencia de Atención Primaria de Tenerife, Primary Health Care San Isidro, 38611 Tenerife (Canary Islands), Spain; 2 Joint Research Centre European Commission, 21027 Ispra, Italy; ecustodio2014@gmail.com; 3 National Centre for Tropical Medicine, Health Institute Carlos III, 28029 Madrid, Spain; 4 Preventive Medicine and Public Health and Microbiology Department, Universidad Autónoma of Madrid, 28029 Madrid, Spain; mrosario.lopez@uam.es; 5 National School of Public Health. Health Institute Carlos III, 28029 Madrid, Spain; bsanz@isciii.es; 6 CIBER Epidemiology and Public Health (CIBERESP-ISCIII), 28029 Madrid, Spain; 7 Nursing Department, Faculty of Medicine, Universidad Autónoma of Madrid, 28029 Madrid, Spain

**Keywords:** exclusive breastfeeding, midwife, primary healthcare, Spain

## Abstract

(1) The objective of our study is to determine, from a primary care midwife’s perspective, which biopsychosocial factors can favour or be detrimental to exclusive breast feeding. (2) The study was carried out in Tenerife (Canary Islands, Spain) and is based on qualitative methodology. Twenty in-depth interviews were carried out with midwives working in primary care centres in Tenerife, using a content analysis approach. The transcript data was then encoded following an inductive approach. (3) According to the perceptions of the primary care midwives who were interviewed, the barriers and facilitators that influence exclusive breastfeeding related to the biopsychosocial spheres of women are, at an individual level, the physical and emotional aspects during the postnatal period; at the relationship level, the presence or not of support from the close family and partner; at the community level, the environment and social networks the new mothers may have; and at the work level, characteristics of jobs and early return to work. (4) The findings of our research can help healthcare professionals to approach the promotion and encouragement of exclusive breast feeding at each of the levels studied, with the aim of increasing rates following recommendations issued by The World Health Organization.

## 1. Introduction

The World Health Organization (WHO) promotes breastfeeding (BF) for at least two years as the unequalled method of providing the ideal food for the healthy growth and development of infants. It also recommends BF to be exclusive, that is, for the infant to have only breastmilk and no other liquids or solids, not even water, for the first six months of life [1].

The WHO recommendations are grounded on the multiple benefits of breastfeeding (BF) in the short- and long term that have already been widely described. BF benefits the newborn by providing immunological factors [2] and conferring protection against infectious diseases [3,4], as well as having beneficial effects on the child´s cognitive development and protecting them from becoming overweight and suffering from obesity and diabetes later in life [4,5,6,7,8]. With regards to the mother, the benefits in the short term are related to a better post-partum recovery, clinically [8,9], as well as psychologically and emotionally [10,11,12,13]. In addition, in the long term, it lowers the risk of developing breast, ovarian and endometrial cancer, as well as other diseases [8]. Additionally, the act of breastfeeding confers advantages to both mother and child by promoting the emotional bond [14], as well as representing economic savings by reducing hospital expenses [15]. It has also been shown that several of these advantages are enhanced if exclusive breastfeeding is maintained for six months, as compared to shorter periods of time [1,16].

Nevertheless, and despite the benefits it provides and WHO′s recommendations, overall BF and EBF rates remain below international target recommendations [17]. In Spain, the most recent figures indicate a prevalence of BF of 81%, 76% and 58% in the first six weeks, three months and six months, respectively [18]. EBF rates are even lower, reaching 66%, 53% and 28% at six weeks, three months and six months, respectively [19]. These rates are far below the WHO target of 50% of EBF for at least six months [20].

The rationales for these low BF rates are varied and complex, and do not depend only on the women themselves at the individual level, but there are issues at many different levels. At the individual level they have been related to physical breast problems [21,22,23], the feeling of insufficient milk production [21,24], low maternal motivation [25] and the mother’s low educational level [26,27]. At the social level, the reasons identified have been the absence of a pro-breastfeeding culture [26,28,29], the lack of support from the partner, family or social environment [27,30], the early return to work of mothers [24,26], as well as the lack of social, labour and economic policies that promote it, together with the lack of advertising control policies [24,25]. At the same time, the lack of support of healthcare professionals [24,26] and the presence of inadequate hospital practices preventing early mother-child contact [31,32] have been identified as important factors related to the low EBF rates in Spain.

Thus, in order to increase the BF and EBF rates it is crucial to follow a comprehensive approach that tackles the individual, social, political, economic and health system dimensions, so more support is offered to women at all levels by all actors involved [33]. One of the key players in this supporting role are midwives, as their job is to provide support, care and advice during pregnancy, childbirth and the puerperium (adopted by the Meeting of the Council of the International Confederation of Midwives, 19 July 2005, Brisbane, Australia). Furthermore, their clinical practice allows a continuous and constant close contact with many women throughout their life cycle, which gives them a key role in supporting BF [34,35]. Several studies recognize that healthcare professionals do not have enough training in BF and that it is midwives who have the greatest knowledge and understanding in this topic [35,36,37]. In Spain, particularly, they have the specific competencies in the advice, support and promotion of BF [36,38].

The support midwives provide women with regards to BF has been defined according to two aspects: as a technical expert, where the physical and physiological part of BF prevails, and as an expert partner with a global vision of women in all spheres of life where she is an active participant of her own BF [39]. The literature shows that the most critical moment of BF, where most problems arise, corresponds to the first days after delivery, when the woman returns home after hospital admission. The first two weeks after are key to establishing BF, where the support and care of the professionals, and more specifically the midwife, is not only necessary, but crucial [40,41,42]. In Spain, it is the primary care (PC) midwives who continue the care and monitoring of women after their hospital stay, thus providing them a unique perspective on the positive and negative factors influencing BF and EBF of mothers in that critical period. Therefore, in this paper we aim to assess the barriers and facilitators of EBF in Tenerife (Canary Islands, Spain) from the perspective of PC midwives.

## 2. Materials and Methods

### 2.1. Study Design

The study was carried out in the island of Tenerife that belongs to the Canary Islands Autonomous Community in Spain. Tenerife is the largest island of all the Canary Islands 105 and the most populated of all Spanish Islands, due to increased birth rates and immigration. Regarding socioeconomic data for the first quarter of 2020, the unemployment rate is 19.8% and the employed population is 395.24 (in thousands of people) [43,44]. Considering the geographical definition of “Metropolitan Area/Zone”, Tenerife has 31 municipalities grouped into 11 counties, geographically located in the Metropolitan Zone, where its capital, Santa Cruz de Tenerife, is located, the North Zone and the South Zone. The population is greatly dispersed, which influences the organisation of health services. Most of the population lives in urban areas (832,736 inhabitants), while 71,977 inhabitants belong to the rural environment [43]. Tenerife’s health area has seven specialised healthcare centres and 39 Basic Health Zones (BHZ) with 101 115 healthcare centres, of which 39 are Health Centres (HC) and 62 local practices [45] (Table 1).

The current PC midwife staff in Tenerife is 53 (52 women and one man).

We performed a qualitative study based on individual in-depth semi-structured interviews, a research method that allows to learn depth, detail and individual perspectives of complex realities [46]. We recruited PC midwives using a convenience snowball sampling technique designed to include a pre-defined set of midwives′ profiles. These included different midwives’ characteristics such as age, workplace, type of population served, work experience, specific training in BF, whether they had children and whether their children were breastfed (See details in Appendix A), in order to adequately represent the point of view of PC midwives in Tenerife and achieve saturation (the point at which no fresh data is evident). One of the researchers (SLLP) contacted the initial key informant, an experienced midwife in charge of coordinating all the BF groups in the island, who referred her to the rest of the subjects, as defined in the snowball technique.

The interview guide or script was developed by two of the authors (SLLP and LOG), and is included in the attachment (Appendix B). With the first eighteen interviews saturation was achieved, but two additional interviews were conducted to fulfil the pre-defined set of profiles. A total of twenty in-depth interviews (20 out of the 53 PC midwives of Tenerife) were carried out with midwives working in PC centres in Tenerife, 13 in the Metropolitan area, three in the North area and four in the South area. Among the interviewees there were midwives working for urban and rural populations, with an age range between 27 and 63 years old and varied working expertise and BF training, as well as mixed motherhood experiences (Appendix A).

### 2.2. Data Collection

The study was presented at one of the monthly meetings held by the PC midwives in Tenerife. They use these meetings to present topics of common interest, update protocols and conduct continuous training courses.

The field work was carried out between the months of November 2018 and February 2020. The interviews were carried out by SLLP according to availability of the midwives in the following manner: Three in person, one by phone call and 16 by video call.

### 2.3. Ethical Considerations

The interviews were digitally recorded in audio after receiving the participants′ written consent. Participants were informed about the objectives of the study and were guaranteed anonymity and confidentiality when expressing their opinions. They were assured that their participation was voluntary and that they could withdraw from the study at any time (Appendix C).

This study obtained prior permission from the Tenerife PC Management (Research Area) and approval of the Research Ethics Committee of the Autonomous University of Madrid (Madrid, Spain).

Throughout the article, we refer to the term “midwife” to refer to both women and men in order not to identify the speech made by the only man who is part of the sample.

### 2.4. Analysis

Interviews, carried out in Spanish, were transcribed verbatim. Data was anonymised prior to performing the analysis and participants′ names were removed from the transcripts and replaced by numbers. The transcripts of the interviews were analysed using a content analysis [47]. To facilitate the coding process we used the programme Open Code 3.6 [48]. Firstly, transcriptions were coded line for line, following an inductive approach that creates emerging codes that summarise the content of each sentence in a paragraph. After, codes were categorised according to whether they were, in general, “facilitators” or “barriers” of EBF, to later identify them in a chronological order by subcategories.

**Figure 1:**
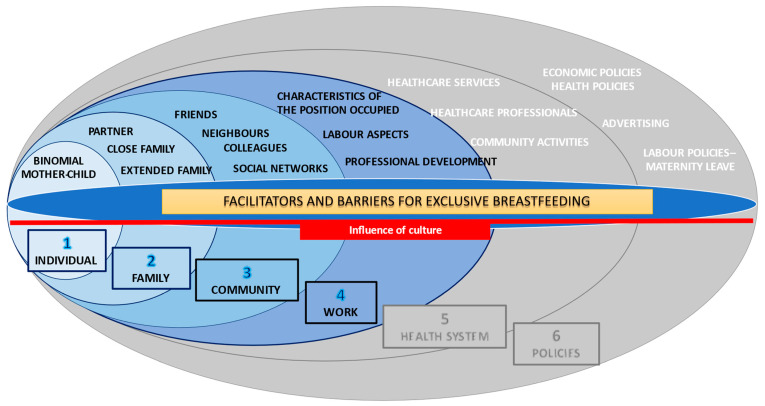
Barriers and facilitators for exclusive breastfeeding in women′s biopsychosocial spheres according to primary care midwives in Tenerife (Canary Islands, Spain). Authors: Seila Llorente-Pulido; Estefanía Custodio; María R. López-Gimenez; Belén Sanz-Barbero; Laura Otero-García. Article Type: Original Research. Adaptation of Bronfenbrenner’s Ecological Model.

This research has its own conceptual framework (Figure 1), adapted from the Ecological Model of Bronfenbrenner [49], where the different levels that affect EBF are indicated, together with the results obtained per level. This work presents only the results related to the first four categories: individual, family, community and work.

## 3. Results

In this section we describe the results for each of the biospheres (individual level, relationship level, community level and work level) following the conceptual framework structure.

### 3.1. Individual Level

#### 3.1.1. Maternal Factors That Make Adherence to EBF Difficult

1.1.1. Physical and physiological factors that lead women to stop EBF.

Midwives perceive that the most common physical problem encountered by women is related to an inadequate latch of the infant to the mother’s breast. This leads to a series of problems that begin with the appearance of cracks causing pain in the nipple, insufficient stimulation of the breast and therefore poor milk production, weight loss of the infant, and, finally, termination of EBF if this is not resolved early.

*“... the first few days the problem they find the most is the latching on, which means they are already starting to have problems with cracks, a chain that can lead to significant suffering due to the pain.” (E7)*

1.1.2. Women feel tired to carry out EBF as part of the upbringing.

Midwives point out that women decide to abandon EBF due to the lack of rest that it entails and the feeling of dependency that looking after a newborn implies. The informants point out that, in the majority of cases, women bear all the weight of the caring tasks and domestic chores. This produces an overload for her and an accumulated fatigue, leading to her decision to stop BF.

*“...as a difficulty after tiredness, lack of sleep... this can be the cause of abandonment, or the lack of will to breastfeed...” (E4)*

1.1.3. The vulnerability of women during the postpartum period harms EBF.

Midwives are aware that the postpartum stage is a period where women experience physical and hormonal changes, but also changes on a psychological and emotional level. The puerperium is a period where women feel vulnerable and insecure in facing this new stage and the great change to their life in general, and BF in particular, that raising a child entails.

*“...because postpartum is the most important period for EBF to be well established and when women are most vulnerable and need support...” (E9)*

1.1.4. Women who lack motivation abandon EBF.

Midwives point out that women’s lack of motivation for EBF is an important factor for its completion, which can be understood when women, prior to childbirth, are unclear about undertaking BF, are not previously informed about it or do not seek help when they have difficulties. The decision to stop EBF can occur at the beginning, before having established BF, or later on, when complications arise in the short- or long term.

*“... if the motivation is not very clear, if you are not sure, difficulties appear and you don′t try to solve them, because the logical thing is that you quit.” (E5)*

1.1.5. Lack of confidence as a “mammalian animal” limits women’s adherence to EBF.

Midwives perceive that women have many doubts and fears about their ability to breastfeed due to the multiple myths surrounding BF. The informants mention that women forget they are mammalian animals with enough capacity to feed their young with only their breast milk. It is this lack of confidence in their own BF, midwives point out, that can hinder their adherence.

*“... We lack a lot of self-confidence, starting during pregnancy, to think that women have milk, they question whether they will have milk, and who doesn′t have milk? I think we have little instinct to follow the natural process...” (E8)*

1.1.6. Ending EBF for artificial feeding as the latter can be delegated.

Midwives perceive that women quit EBF because they consider that artificial feeding provides greater comfort by providing more rest, greater ease in delegating infant care and the possibility of being able to lead a full social life.

*“...we live in a culture of social comfort, where you want things to be solved now and at this very moment, and of course breastfeeding is not from that comfort culture.” (E7)*

1.1.7. Women quit BF if they have had close negative experiences.

Midwives indicate that women with previous negative experiences in BF, either their own or through experiences of other women they are close to, such as their sisters, friends, etc., already decide to use artificial feeding when they are pregnant.

*“...there are women who may have had previous negative experiences or a friend of theirs had a horrible experience, so they are already going downhill and she says: no, I’m not going to nurse...” (E2)*

#### 3.1.2. Maternal Factors Facilitating EBF Adherence

1.2.1. The predisposition of women to carry out EBF.

Midwives point out that the puerperium is a critical moment in which women experience changes on a physical level, but above all on an emotional level. The predisposition of women towards EBF is, according to them, the main driver to overcome the possible difficulties that may arise and finally make it successful.

*“The motivation that each woman has to breastfeed and their conviction; that is the main positive factor for adherence to exclusive breastfeeding.” (E1)*

1.2.2. EBF as a satisfying and empowering bonding experience.

Midwives describe how women tell them that being able to breastfeed produces satisfaction and well-being. The hormones secreted during EBF facilitate the establishment of emotional ties as a biological survival instinct, favouring the bond between mother and child.

*“... the most important thing they say is that they know that breastfeeding is the best food there is, they are helping their defence system, and it is satisfying for them to be able to breastfeed their baby and also for the bond they have with the baby.” (E12)*

1.2.3. The importance of previous positive experiences of BF.

The informants perceive that women who have already had at least one child, and have a previous positive experience with EBF, find it easier to carry it out. The confidence in themselves is greater as well as the tools to solve possible problems that may arise.

*“The first child opens the way and, more than anything, you already know how to solve difficulties and I believe that women are no longer so overwhelmed, since when the baby cries they simply put the baby to the breast without questioning themselves too much. They are more confident.” (E5)*

1.2.4. The active search for information in EBF as an indicator of greater adherence.

Midwives emphasise the importance of women informing themselves so they acquire the knowledge and tools that allow them to better understand the dynamics of EBF, thus increasing their self-confidence.

*“...the more information you have the more power you will have later, especially during labour, about your delivery and about your lactation, the more information the better...” (E14)*

1.2.5. Socioeconomic and cultural level and EBF.

Midwives perceive that women with a higher socioeconomic and cultural level have higher rates of EBF because they have the tools and skills to search for adequate information. Furthermore, these women have a greater ability to discern the information with scientific evidence.

*“…women who have a higher economic level, with a university degree, and have a job have more access to information, and they are better informed and perhaps they have more initiatives and more desire to breastfeed than others.” (E4)*

### 3.2. Relationship Level

#### 3.2.1. The Family as a Barrier for EBF

1.1.1 The partner′s lack of support impairs EBF.

Midwifes indicate that the lack of support from the partner is a negative factor for EBF. This lack of support can mean a lack of implication in the upbringing because the partner believes EBF is not worth the sacrifice it implies for the woman and they count on artificial feeding as an affordable solution.

*“... If you do not have the support of your partner and you have a person next to you who continues doing a bit their own thing, without getting involved, then even worse because you have to continue doing everything you did before plus breastfeeding…”* *(E14)*

1.1.2. Experience in parenting endorses the mothers of the mothers/mothers-in-law to advise artificial feeding against BF.

Midwives point out that women quit EBF due to lack of support from the extended family, specifically from the mothers of the mothers/mothers-in-law, who recommend artificial feeding, the same as they did with their own children. They show the new mother they have more experience and capabilities to look after the baby than she has, and she follows the advice received.

*“…and particularly the lack of support by the mothers or mothers-in-law, who didn′t breastfeed their babies. It is what they did and what they recommend.” (E17)*

#### 3.2.2. The Family as Facilitator for EBF

1.2.1. The partner as a fundamental pillar for a successful EBF.

Midwives highlight the important role the couple plays in the success of EBF in particular, and they describe their role as key to support the woman in such a vulnerable time as the post-partum period. Particularly if there is a distribution of household and upbringing tasks, this helps women breastfeed.

*“If your partner is your accomplice in that support, also as a caregiver of the baby, that influences you, it will help you in breastfeeding, because he helps you in sharing what parenting is. Mothers who have support from their partner, I think they achieve a more successful and longer lactation.” (E7)*

1.2.2. The grandmothers who EBF support women, thus promoting adherence.

The informants highlight the importance of support from mothers/mothers-in-law of the women (grandmothers) in EBF, in particular of those who decided to EBF at a time when artificial feeding was recommended. Midwives consider the grandmother as a benchmark in EBF for women and the second most important support figure after the partner.

*“…When your mother, your grandmother and your great-grandmother have breastfed, it is very rare that you do not breastfeed because they will be on top of you. She feels she doesn′t want to be less than them, you know?” (E11)*

1.2.3. The extended family favours EBF by collaborating in the distribution of domestic and care tasks.

Midwives indicate that the family that supports EBF collaborates in the distribution of household chores and the care of the other children they have, allowing the woman to spend more time caring for the baby and their EBF.

*“…The mother and the mother-in-law help in the postpartum providing their home-cooking in tuppers and taking care of the baby so that they can shower and stuff. The family is a very important positive factor for recovery and for maintaining lactation.” (E2)*

1.2.4. The close presence of lactating women favours EBF.

Midwives point out that the positive experience of close relatives in EBF is very beneficial because the woman feels supported.

*“...it is important for breastfeeding to have someone who has breastfed in your close circle, they may be mothers, grandmothers or an important figure such as a sister...” (E1)*

### 3.3. Community Level

#### 3.3.1. An Environment against BF as a Barrier for EBF

1.1.1. The setting does not support EBF.

Midwives point out that the lack of support from the setting, with negative comments about BF and the way of parenting, can create anxiety and burden mothers, leading them to decide to stop EBF. It is not easy for women to repeatedly have to endure, from their surroundings, a constant questioning about their way of caring for their baby.

*“... if you want to breastfeed, but above all your environment is hindering you and they are continuously giving you a negative message, particularly at that very changing postpartum period, it is very easy to succumb.” (E14)*

1.1.2. Women stop EBF because they decide to continue maintaining their previous social life.

The informants perceive that women want to continue leading the same social life as before, such as going out with friends or family, taking trips, etc., and this is sometimes incompatible with EBF. BF leads women to change the way they interact with others.

*“Wanting to continue living the life you had before, the same trips, the same leisure, but breastfeeding puts a hold on this in many respects, because you can’t handle everything.” (E13)*

1.1.3. Gender inequality negatively affects BF.

In Table 2 we have compiled the midwives’ perspectives on this issue.

1.1.4. A society that carries a culture of artificial feeding harms BF.

Midwives point out the difficulty new mothers encounter when deciding to BF in a society like the Spanish one, where, in the 1930s, artificial feeding was the norm. All the myths and beliefs around the feeding of newborns often become the justification for abandoning BF and choosing artificial feeding as being healthier.

*“… It is still very damaging that we are still dealing with women who have suffered the pressure of not breastfeeding and using formula. We know the great pressure and the great business that existed at that time.” (E3)*

1.1.5. The current social construction of motherhood can negatively affect EBF, as reflected in Table 3 more in detail.

#### 3.3.2. A Protective EBF Surrounding Facilitates Adherence

1.1.1. Women who are breastfeeding seek physical and/or virtual support networks to continue EBF.

Midwives point out that breastfeeding women seek support networks to accompany and share their experiences. The search for support occurs through social networks or through the creation of BF groups. The informants refer to these networks as “tribes”.

*“... There are many women who breastfeed and follow midwives who have webpages, or mothers who breastfeed a lot and a social network bond between these women, that perhaps didn* *′* *t exist in society, is formed.” (E8)*

1.1.2. Women belonging to pro-breastfeeding cultures choose EBF.

The informants point out that there are cultures that are more predisposed to breastfeeding and it is more common for BF knowledge to be passed from generation to generation, making it normal and more visible as the ideal food for the baby.

*“... African or Arab women have very few breastfeeding problems, they have seen it with their sisters, or have several children and live in a community, they help each other.” (E9)*

1.1.3. Living in a rural area improves women′s adherence to EBF.

Midwives point out that women who live in rural areas decide to EBF more than in urban areas since they live closer to the extended family and have a more nurturing environment where EBF is the usual way to feed children.

*“...society in rural areas sees breastfeeding more normal and they do it naturally, different to what happens in city areas. In the villages they have more of a habit of breastfeeding.” (E14)*

### 3.4. Work Level

#### 3.4.1. Labour Factors That Harm BF

In Table 4 we have assembled the midwives’ perspectives on work characteristics that hinder EBF.

#### 3.4.2. Work Factors That Promote EBF

We have not found any work factors that promote EBF. The lack of work facilities to maintain EBF leads women to overexertion.

Instead, we have reflected in Table 5 the motivations and strategies to reconcile work and family life as described by the midwives.

**Figure 2:**
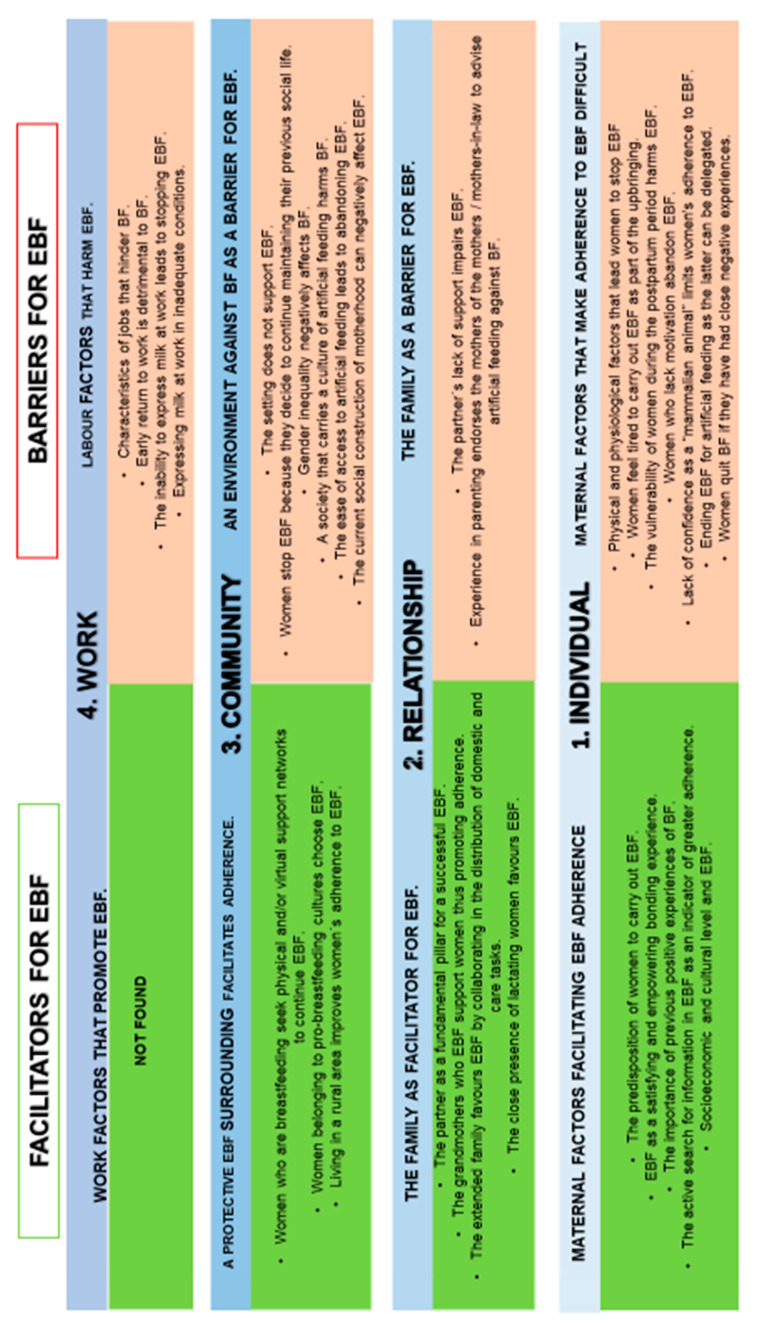
Facilitators and barriers for EBF in relation to the biopsychosocial sphere of women.

In Figure 2 we represent a summary of the results included in each section.

## 4. Discussion

PC midwives of Tenerife describe, from their point of view, the different factors that positively and negatively influence EBF in relation to the biopsychosocial sphere of women at the individual level, relationship level of the couple and family, community level of the environment and social networks, and at the work level.

In our study, at the individual level of women, midwives perceive that the barriers are related to physical and physiological complications in the breasts associated with an inadequate latch by the baby leading to pain and cracks, as well as other problems, such as breast engorgement and mastitis. This negative factor is also discussed by Medina et al. (2013) who relate the ineffective latch to the nipple to an inadequate nursing technique leading to the appearance of alterations in the breast [22]. Midwives point out that a low level of training negatively influences BF, as reflected in the study by Oribe et al. (2015), where they claim that the majority of women do not know how to prevent and solve these problems [21]. Calvo-Quirós (2008) relates maternal insecurity with a lack of knowledge about BF [50]. This maternal insecurity is of particular importance in the postpartum period, a period of increased vulnerability according to midwives, which specifically leads to women′s lack of confidence and inability to feed their child, facilitated by the feeling of unreal hypogalactia. Oribe et al. (2015) and Medina et al. (2013) in their systematic reviews address the physical problems that can appear in the breast due to the lack of confidence and security in EBF, which is acquired through knowledge and information provided by healthcare professionals [21,22]. Medina et al. (2013) recognises that there is no consensus regarding the research methodology to relate the early breast problems with an inadequate technique. Midwives in our study emphasise the importance of reassuring mothers about their “mammalian” condition [22].

Another barrier at the individual level that midwives in our study highlight is the fatigue perceived by women, during the postnatal period, that negatively affect EBF and lead to its cessation. Quevedo-Navarro et al. (2014) define it as postpartum fatigue and describe it as “an imbalance between activity and rest, which hinders the well-being of the affected person” [51]. Along these same lines, Díaz-Gómez et al. (2016) mention night time awakenings as a reason leading to BF abandonment, since artificial feeding can be delegated and BF cannot [24]. Past negative experiences in BF, both one′s own and those of others are also seen as having a negative influence on EBF initiation and duration. This negative experience is transmitted within the family from mothers to daughters, causing rejection of BF, as seen in other studies [52].

In order to turn around the situation the midwives indicate that, on an individual level, the women’s motivation to keep BF is essential. This motivation may have different origins. According to our study, midwives perceive that women with a high socioeconomic and cultural level have a greater predisposition to breastfeed and therefore higher rates of EBF, also found by other authors [24,26]. Specifically, González et al. (2008) found that higher education levels are related to higher rates of BF, possibly due to the ease of access to information [27]. On the other hand, midwives also indicate that the satisfactory experience breastfeeding produces in women is a positive factor, as seen in the study by Thomson et al. (2012) [25]. There is scientific evidence that shows that the well-being produced by breastfeeding prolongs EBF, and is associated with a higher quality and duration [24,26]. Rius et al. (2014) refer to the subjective feeling in the mother when she realises she is capable of producing enough milk and is able to feed her child, which increases her self-confidence [26]. These results reinforce the importance of providing women with support and training, as well as promoting BF in order to overcome physical and physiological difficulties with BF and build up the mothers′ motivation to initiate and continue with appropriate BF practices during the recommended time periods. Therefore, we recommend the design of activities aimed at promoting BF from pregnancy and childbirth that offer theoretical and practical information on the proper breastfeeding techniques and how to address possible BF problems or complications, which would motivate women as well as improve their self-confidence.

Moreover, the midwives also point out the importance of assistance from the partner and close family for the maintenance of EBF, coinciding with the mother’s perspectives described in the studies by Conde-Puertas et al. (2014), where the women who had been interviewed indicated that their partner was key to support in BF [53], followed by the support from grandmothers, both the mothers and the mothers-in-law of the women [24,26]. In their systematic review, Ogbo et al. (2020) have studied the active support activities that partners can carry out in order to favour EBF, according to women′s needs through not only verbal stimulation but collaborating in house chores and with the upbringing of children [54].

The same way as the partner′s and family′s support can be a facilitator of EBF, midwives also highlight the lack of this support as an important barrier to EBF, similar to what was found by González et al. (2008) [27]. We thus recommend that the BF training and promotion activities are also targeted to the partner and the rest of the family particularly to the mothers of the mothers/mothers-in-law, so as to increase their involvement and understanding of the mother-child binomial, and therefore encourage the BF culture.

Furthermore, women who experience (ex) partner violence during pregnancy, childbirth and postpartum are more vulnerable, as they frequently suffer from mental health disorders, more cardiovascular problems and increased substance use [30,55,56,57]. Thus, they have increased difficulties in carrying out their self-care and suffer more insecurities when caring for their child and therefore maintaining BF [58]. In fact, it has been observed that pregnancy and postpartum are the periods where (ex) partner violence intensifies, with psychological violence being the most prevalent [55,59,60], and negatively affecting BF [58].

Other BF barriers at the community level identified by the midwives are related to the lack of support from a society where the bottle culture predominates. This finding coincides with that found by other authors [53], indicating a negative relationship between duration of BF and little social support. In our study, midwives point to social demands as a negative factor since, for women, BF is a barrier to maintaining previous social relationships, as seen in other contexts [61].

Conversely, a supportive culture, environment and/or social network positively affects breastfeeding practices. Midwives interviewed report the existence of practices and beliefs belonging to certain pro-breastfeeding cultures where women find it easier to breastfeed than others. This finding can also be seen in other studies [26]. Otal-Laspaus et al. (2012) explain that, in many cultures, BF is considered a fundamental part of the upbringing and thus women find it easier and have more support to breastfeed their children [62]. Midwives also describe the differences between urban and rural areas, with most reporting greater adherence in the latter. This may be related to the maintenance of traditional practices and a more supportive environment in the rural settings as indicated in the study by Calvo Quirós (2009) [50]. We thus recommend the creation of networks of pregnant women in the health centres themselves, and offering adequate virtual tools, where women can go and feel protected.

Additionally, the midwives highlight the importance of advertising in favour of BF and propose a change in perspective, with advertising campaigns showing active mothers breastfeeding, and not only mothers in the intimacy of their homes or bedrooms. Along these same lines, Díaz-Gómez et al. (2016) point out the advantages of breastfeeding promotion campaigns where the benefits of breastfeeding are emphasized, even if the disadvantages of artificial lactation are not mentioned [24].

At the work level, the midwives in our study did not identify any BF facilitators but highlighted several barriers. They underlined the negative relationship between current maternity leave in Spain and the duration of EBF. They indicate the return to work as one of the most critical moments for EBF, addressed also by other authors [21,24,26,27]. A maternity leave of less than six months is one of the main reasons for ending EBF [27]. Muñoz (2008) indicates that the precarious working conditions and the few possibilities to maintain BF lead women to end it [63]. On the other hand, midwives also point to the difficulty and lack of support women have to express milk during the working day, making it difficult to stimulate milk production due to stress [63]. In fact, Díaz-Gómez et al. (2016) states that stopping BF is more related to the low production due to not being able to express milk during working hours, than the actual return to work [24].

Lastly, midwives mention the efforts and sacrifices women have to make in order to make EBF compatible with work, since in Spain there are few possibilities and legal resources to delay the return to work. In this respect, as reflected also in the study by Muñoz (2008), midwives highlight the importance of family support so women can go back to work [63]. Our recommendation is the design of strategies and public policies designed to promote BF and the work life balance, prolonging the maternity leave so EBF can be guaranteed for the first six months of life and that this period is remunerated.

### Limitations of Our Study

Due to the study design, we are unable to establish a hierarchy in the results, that is identify which are the most important factors or those that most affect EBF, but we provide a rich description of this complex reality instead.

The convenience sampling hinders the possibility to generalize the results of the survey to the population as a whole, but we consider that the snowball technique applied allowed to saturate the discourse of the PD midwives according to their different profiles.

On the other hand, the use qualitative methods do not allow the results obtained to be extrapolated to other populations, although they provide in depth and detail perspectives not feasible to be obtained by quantitative methods. The health, labour and social policies, as well as pro-breastfeeding culture are heterogeneous and dependent on the context, limiting the generalisation of our conclusions across settings.

## 5. Conclusions

The facilitators and barriers for EBF in the island of Tenerife (Spain) happen at different levels: at work, community, relationship, as well as the individual level. Thus, revealing that the success or failure of BF is not only related to the mothers themselves, but instead, is an issue that needs to be addressed at all levels.

This study provides policy recommendations at each of the levels identified. We thus consider it can help health professionals to approach BF interventions, improving the information, assistance and support of mothers, their partners and their children, and it can also support policy makers to design informed policies that address the problem with an integrative perspective.

## Figures and Tables

**Table 1:** Inhabitants according to the area of the island, taking into account foreign population (ISTAC 2018), employed population (ISTAC 2020) and BHZ (https://www3.gobiernodecanarias.org/sanidad/scs/mapa) (accessed on 26 May 2020).

	North	South	Metropolitan	Total Population
Number of inhabitants	224.047	291.706	388.960	904.713
Number of foreign inhabitants	14.218	81.875	21.103	117.196
Employed population (in thousands)	93,42	129,78	172,04	395,24
Number of BHZ	12	10	17	39

**Table 2:** Gender inequality negatively affects BF.

Subcategories	Quotes from the Midwives
**The negative influence of male chauvinism on upbringing and BF.** Midwives perceive the negative influence of male chauvinism on upbringing and BF. Socially, there are many cases in which sexualisation of women occurs where women are valued more for their physical appearance than for other qualities or capacities. This generates an increased pressure and anxiety for them to continue staying “pretty or beautiful” in the same way as before.	*“...on a social level women are valued above all for their physical appearance, so we are immediately concerned with being as we were before, beauty and consumption are the things that are valued and it is not valued that a woman breastfeeds for three years, these women are referred to as hippies or weirdos…” (E8)*
**The gender inequality in the couple relationship.** The informants perceive that this gender inequality also occurs in the couple relationship, becoming compromised when they become parents. The woman focuses on caring for the child and the partner feels neglected, receiving less attention than before.	*“…There are many couples who separate in the postpartum period. The couple is tested, in the change from a couple relationship to parents of a baby”. “And many times, they do not support them in breastfeeding, on the contrary, they complain that they do not have time for them.” (E4)*
**The simplistic conception of the woman’s breast.** As another negative factor for BF in relation to the chauvinist society, the midwives point out the simplistic conception of the woman’s breast as an element without biological function, by not respecting BF in public. Socially, the breast of the woman in its breastfeeding function is not as accepted, despite the fact that BF in other mammals is more normalized.	*“...censuring a woman who is breastfeeding, because she′s showing her tit. Many women breastfeeding in public get bad looks, there are even people who tell them to cover themselves…” (E2)*

**Table 3:** The current social construction of motherhood can negatively affect EBF.

**The loneliness that accompanies motherhood.** Midwives highlight the feeling of loneliness that characterises motherhood today. They explain that before, women lived with the family and had the opportunity to face this stage together, while currently many women do not have the support of their partner or family, because they are not physically close or because their presence and involvement in parenting is non-existent. In these cases, women feel alone in seeing to the needs of a newborn 24 h a day, where additionally BF requires more time and exclusive dedication, without the possibility to delegate and be able to do other things.	*“...the feeling of loneliness I think is an important factor, which is ascribed to motherhood, we women have it now in the postnatal period and it is seen more or less intensely depending on the support you have.” (E1)*
**Being a mother: a clash between expectations and reality as a negative factor for EBF.** Midwives point out that the social idealisation of motherhood makes it difficult for women to adapt to the new situation. The lack of close references and true information on postpartum and motherhood causes women, after childbirth, to find themselves in a situation they did not expect, and without any tools to face it.	*“...the differences between fiction and reality. The imaginary idea they had of what it is to be a mother, what they′ve seen in movies, what they′ve seen with their friends, a couple of hours is not the same as 24 h in the leading role of a nursing mother.” (E15)*
**The current individualistic society: loss of the “tribe” sense, key to EBF.** Midwives highlight the influence of the general functioning of today’s society, much more individualistic in upbringing and especially in BF. The informants perceive that it can be a negative factor for new mothers to not have any close lactating women as a reference and as an example for them.	*“We are in an individualistic society, raising our child alone, so you spend a lot of time alone with your baby in your house taking care of it without other women who support you, we are not surrounded by our relatives who support us with breastfeeding as they used to.” (E4)*

**Table 4:** Work characteristics that harm BF.

**Characteristics of jobs that hinder BF.** Midwives indicate that women find it difficult to maintain EBF due to the lack of support at work in reconciling it with family life, related to the characteristics of the position they occupy.	*“...women with important positions do not even have half an hour of rest, but must always be available. This is causing them a lot of anxiety in returning to work, so they decide to bottle-feed.” (E15)*
**Early return to work is detrimental to BF.** Midwives perceive that insecure working conditions determine an early return, hindering EBF and upbringing.	*“…The population that I attended had very difficult work contracts, if they did not come back within 6 weeks, they no longer had a job. The commitment to their job and job insecurity, had a great influence on that too...” (E3)*
**The inability to express milk at work leads to stopping EBF.** The informants point out that the inability to express milk during the working day causes discomfort and problems that force women to abandon BF.	*“...especially the main problem is that not all jobs allow you to go and express your milk. Some mothers have told me: ′Look, I have to stop this because throughout the day I could not go at any time to express milk and my breasts hurt, I could not stand it.” (E14)*
**Expressing milk at work, but in inadequate conditions.** Midwives indicate that women manage to express milk at work in order to continue their BF, but in inadequate conditions, using their resting time for it, as in many jobs it is not even contemplated. They do not even have a dedicated space for it, and milk is usually expressed in bathrooms or remote places, where women feel uncomfortable.	*“...There are women who use the half hour they have for breakfast to express milk or there are times when they feel strange or are embarrassed. Sometimes they cry in the bathroom.” (E17)*

**Table 5:** Motivations and strategies to reconcile work and family life.

**Maternal motivation is a protective factor to continue breastfeeding after returning to work.** The informants point out that the return to work is a moment of important crisis in EBF. Many women highlight that the degree of involvement and motivation for EBF is more important than the return to work itself.	*“...in the end, those who really believe in breastfeeding, the fact that they work or not, is not decisive.” (E15)*
**Working woman and mother, conflict of interest.** Midwives emphasise that the return to work with a small baby implies a conflict of interest for women, where in one way or another it is a resignation, either at a professional level or at the level of more present parenting.	*“Well, many times you give up breastfeeding or maternity or a more present upbringing you would like, the neuropsychology supports it or you have to leave your job a bit. Sometimes they are forced to leave even their job or a job promotion…” (E1)*
**Women seek ways to reconcile EBF and work at the cost of pay cuts.** Midwives find that women are looking for a way to reconcile EBF but many times that implies a reduction in income or an increase in expenses if they decide to take the baby to a nursery.	*“And if I reduce the working hours, on the days that I do have to work, I have to pay for childcare, then I earn less and on top of that I am paying for care...” (E3)*
**Need to plan ahead and get used to it previously.** Midwives perceive that women are very concerned with going back to work and the care of their child in their absence, so many prepare for that moment. Women decide to plan and start bottle-feeding or anticipate the introduction of complementary foods so someone else can feed their child while they are at work.	*“… If my child is going to stay with my mother or is going to go to kindergarten, I start giving him a bottle when he′s two months old so he can get used to it, it is like planning ahead…” (E8)*

**Figure A1:**
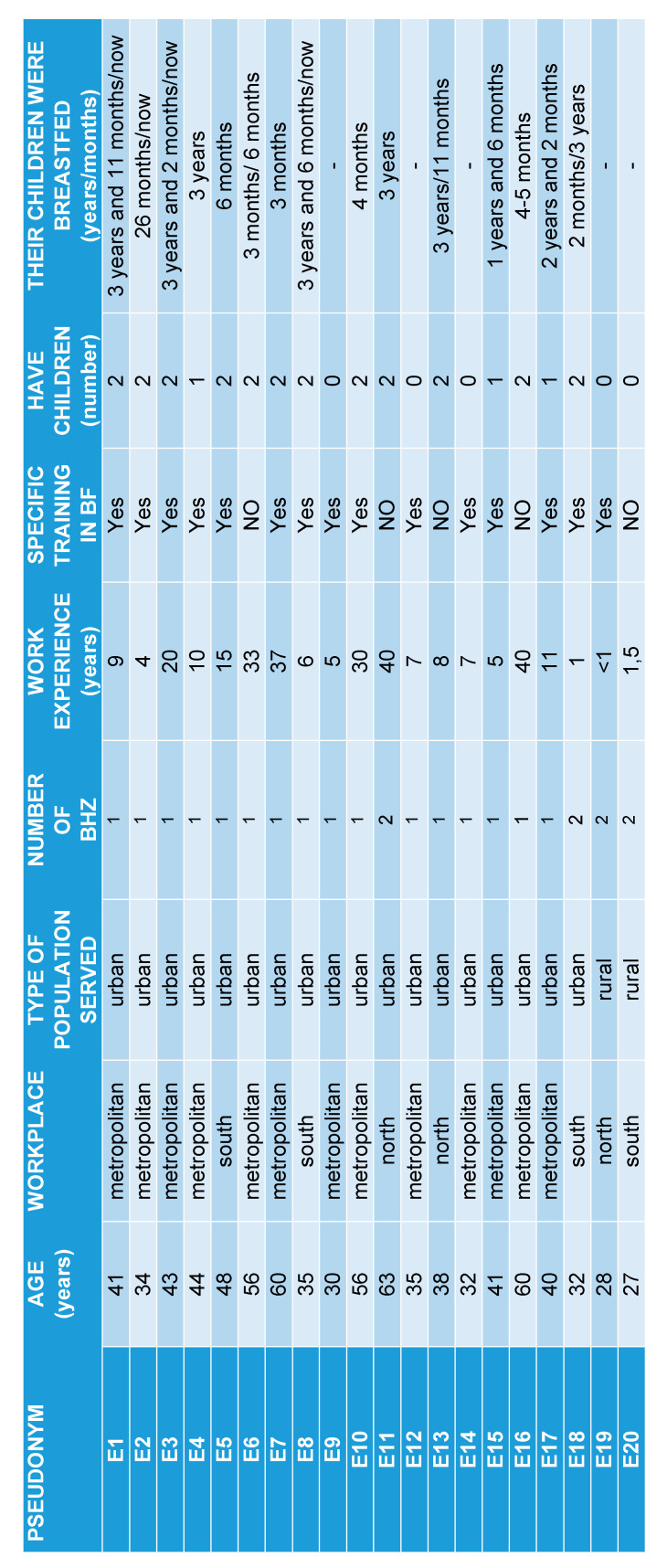
Profiles Primary Care Midwives.

**Figure A2:**
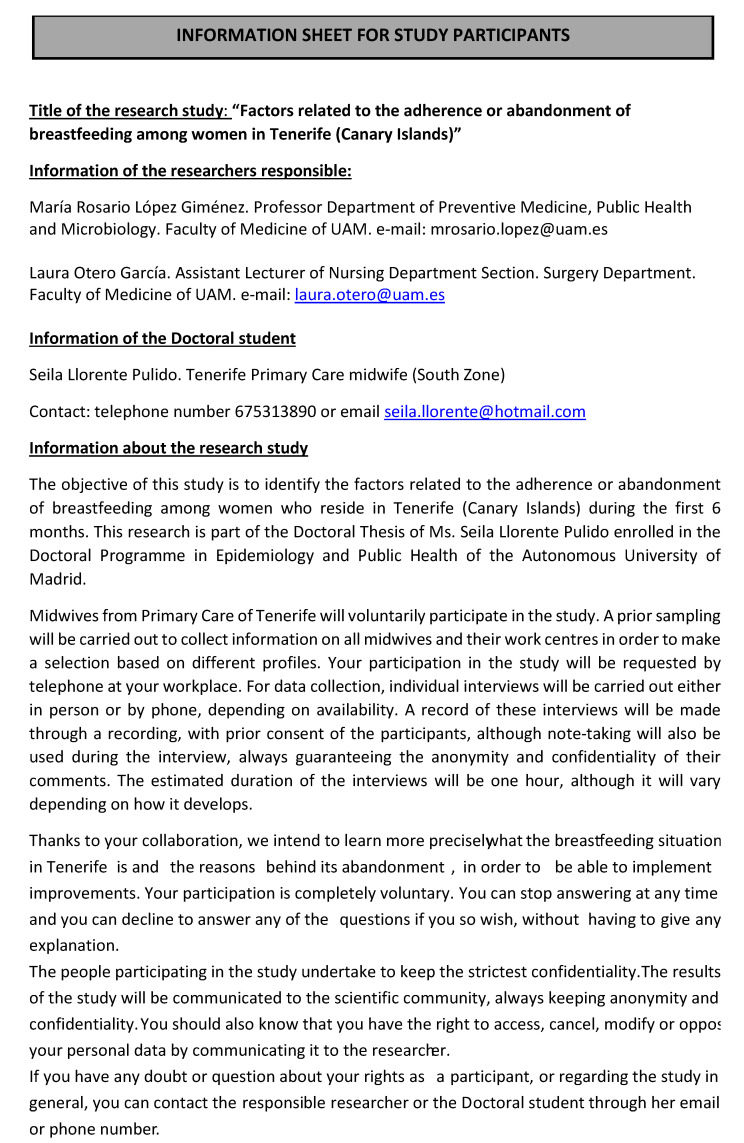

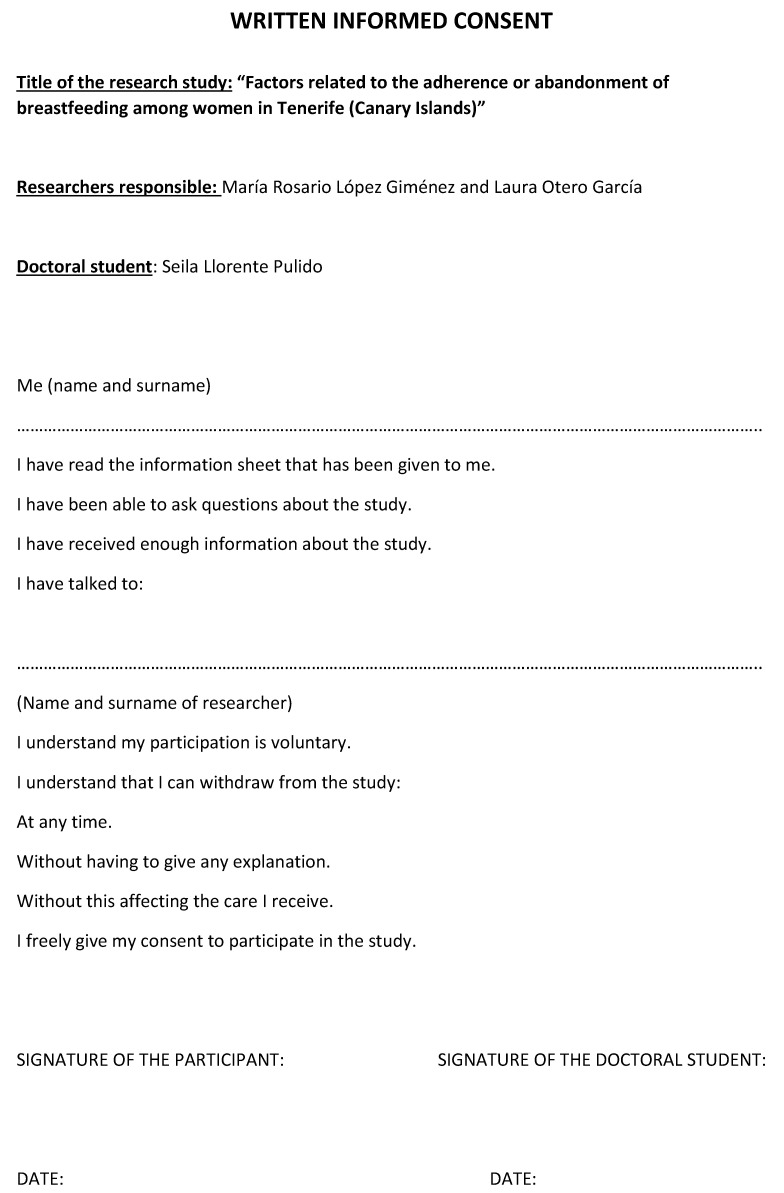
Information Sheet and Informed Consent.

## Appendix B. Interview Script for Midwives

1. **Facilities for women to breastfeed.**

Based on your experience in consultation, what facilitating aspects do women verbalize when breastfeeding?

Do they have support at the family level? Do they have support from their partner? Do they have the ability to continue breastfeeding while at work?

2. **Barriers or obstacles women have to breastfeed.**

Based on your experience in consultation, what barriers for breastfeeding do women verbalize?

What are the most common problems women encounter during the first days when breastfeeding?

What are the most common problems women encounter when breastfeeding once breastfeeding has been established?

At what point do they have the most difficulties: at the beginning or in the following months?

Do they have breastfeeding problems after returning to work? What do women do to reconcile breastfeeding and work?

Where do they more easily express their problems: in consultation or in breastfeeding workshops?

3. **PERCEPTIONS about the causes of breastfeeding abandonment.**

In your experience, what factors do you consider responsible for the abandonment of breastfeeding during the first six months?

4. **PERCEPTIONS about the causes of breastfeeding adherence.**

In your experience, what factors do you consider responsible for adherence to breastfeeding during the first six months?

5. **Proposal to improve the encouragement and promotion of adherence to breastfeeding.**

Currently, what do you do in consultation to promote breastfeeding adherence? What could you do and what resources would you need to improve your care in this regard?

What measures would you propose at the social level to encourage and promote breastfeeding?

What measures would you propose to the health system to encourage and promote adherence to breastfeeding?
